# T2 heterogeneity: a novel marker of microstructural integrity associated with cognitive decline in people with mild cognitive impairment

**DOI:** 10.1186/s13195-020-00672-9

**Published:** 2020-09-10

**Authors:** Alfie R. Wearn, Volkan Nurdal, Esther Saunders-Jennings, Michael J. Knight, Hanna K. Isotalus, Serena Dillon, Demitra Tsivos, Risto A. Kauppinen, Elizabeth J. Coulthard

**Affiliations:** 1 Bristol Medical School, University of Bristol, Bristol, UK; 2 Institute of Clinical Neurosciences, North Bristol NHS Trust, Bristol, UK; 3 School of Psychological Science, University of Bristol, Bristol, UK

**Keywords:** Magnetic resonance imaging, Alzheimer’s disease, Early diagnosis, Ageing, Hippocampus, T2 relaxometry

## Abstract

**Background:**

Early Alzheimer’s disease (AD) diagnosis is vital for development of disease-modifying therapies. Prior to significant brain tissue atrophy, several microstructural changes take place as a result of Alzheimer’s pathology. These include deposition of amyloid, tau and iron, as well as altered water homeostasis in tissue and some cell death. T2 relaxation time, a quantitative MRI measure, is sensitive to these changes and may be a useful non-invasive, early marker of tissue integrity which could predict conversion to dementia. We propose that different microstructural changes affect T2 in opposing ways, such that average ‘midpoint’ measures of T2 are less sensitive than measuring distribution width (heterogeneity). T2 heterogeneity in the brain may present a sensitive early marker of AD pathology.

**Methods:**

In this cohort study, we tested 97 healthy older controls, 49 people with mild cognitive impairment (MCI) and 10 with a clinical diagnosis of AD. All participants underwent structural MRI including a multi-echo sequence for quantitative T2 assessment. Cognitive change over 1 year was assessed in 20 participants with MCI. T2 distributions were modelled in the hippocampus and thalamus using log-logistic distribution giving measures of log-median value (midpoint; T2μ) and distribution width (heterogeneity; T2σ).

**Results:**

We show an increase in T2 heterogeneity (T2σ; *p* < .0001) in MCI compared to healthy controls, which was not seen with midpoint (T2μ; *p* = .149) in the hippocampus and thalamus. Hippocampal T2 heterogeneity predicted cognitive decline over 1 year in MCI participants (*p* = .018), but midpoint (*p* = .132) and volume (*p* = .315) did not. Age affects T2, but the effects described here are significant even after correcting for age.

**Conclusions:**

We show that T2 heterogeneity can identify subtle changes in microstructural integrity of brain tissue in MCI and predict cognitive decline over a year. We describe a new model that considers the competing effects of factors that both increase and decrease T2. These two opposing forces suggest that previous conclusions based on T2 midpoint may have obscured the true potential of T2 as a marker of subtle neuropathology. We propose that T2 heterogeneity reflects microstructural integrity with potential to be a widely used early biomarker of conditions such as AD.

## Introduction

Alzheimer’s disease treatments and therapies that stop or slow down neuropathology will be most effective if administered as early as possible; before significant neurodegeneration has occurred. Accurate early Alzheimer’s disease diagnosis is vital to identify appropriate clinical trial study groups of ‘at-risk’ individuals to expedite development of new compounds [1, 2] and to target disease-modifying treatments when available.

Structural and quantitative MRI show promise in their ability to identify changes in the brain that indicate early Alzheimer’s pathology. Measuring the volume of the hippocampus and entorhinal cortex has been shown to predict progression of mild cognitive impairment (MCI) to Alzheimer’s disease [3–10]. Detectable change in volume is indicative of significant tissue loss, which is likely to be irreversible. As treatment with disease-modifying therapies would be optimal before such significant macrostructural change, we ask whether MRI could be used to identify microstructural changes that occur earlier in the disease-course, before significant volume loss.

Prior to significant loss of tissue volume, several microstructural changes take place as a result of Alzheimer’s disease pathology—(i) oligomers and plaques of β-amyloid (Aβ) and neurofibrillary tangles (NFTs) build up around the medial temporal lobe (MTL) and the thalamus [11–14], (ii) iron is elevated in the brains of people with MCI and Alzheimer’s disease [15] and (iii) even small amounts of necrosis leading to breakdown of cell membranes and oedema will increase the motility of water within a given region. Increase in water motility is not necessarily specific to Alzheimer’s disease and can occur in healthy ageing [16–18]. Accurately measuring such microstructural changes may allow identification of tissue that is at risk of degradation or has reduced functionality compared to a previous state.

T2 relaxometry is an MRI approach that may be able to report microstructural tissue integrity. Relaxation time is a measure, detectable by MRI, that describes the time taken for protons to return to a state of equilibrium following electromagnetic excitation. Specifically, T2 relaxation describes the transverse component of magnetisation. T2 relaxation time of biological tissue varies depending on its physical properties and its surrounding environment. It is primarily driven by water content and mobility and the presence of macromolecular structures and paramagnetic materials, e.g. iron [19–23]. For example, pure water will have a very long relaxation time, whereas in fatty substances, T2 will decay much quicker. T2 is therefore sensitive to microscopic and physico-chemical tissue properties that can change as a result of pathology. Previous research has shown that T2 relaxometry is independent of, and can provide distinct microstructural information to, diffusion tensor imaging metrics [24, 25].

Given that quantitative T2 can be easily measured on routine MRI scans, adding just a couple of minutes to standard T2-weighted structural scanning times, it has been previously explored as an early marker of Alzheimer’s disease pathology. However, previous studies on the effect of Alzheimer’s pathology on T2 in the human brain have yielded varied and sometimes contradictory results. Most studies describe a prolonged T2 in the hippocampus of those with Alzheimer’s disease [26–30], whereas others find the opposite [31, 32] or no change at all [33] (see Tang et al. [34] for a comprehensive review). In two studies [26, 28], change in thalamic T2 was not associated with Alzheimer’s pathology or cognitive impairment, but T2 increased with age [28]. In another study, Dawe et al. [30] found that Alzheimer’s pathology was associated with decreased T2 within the thalamus.

These inconsistencies in human literature are not fully reflected in studies of transgenic rodent models of Alzheimer’s disease, which consistently show a decrease in hippocampal T2 [34]. Inconsistencies within the human literature and between human and animal studies could be a consequence of the multiple pathological processes occurring in the human brain that have opposing effects, either shortening or lengthening T2. In contrast, mouse models are usually dominated by a single pathological process such as amyloid deposition.

Increased water content, such as that caused by increased cerebrospinal fluid (CSF), oedema or cell membrane damage, will prolong T2 [28]. Conversely, increase in iron [22] or in macromolecule-to-water ratio due to accumulation of high density protein aggregates, such as Aβ, shorten T2 [35]. Even in early stages of the pathological progression of Alzheimer’s disease within the brain, factors which cause T2 to either increase or decrease are both occurring in early-affected regions such as the hippocampus and the thalamus [11, 14, 36, 37]. Either effect may be more or less dominant in clusters throughout these regions. Averaging across the entire region could therefore yield, on average, a net change in T2 of zero. A change in the average value of T2 would only come about if T2-shortening factors dominate over T2-prolonging factors or vice-versa, which may not be the case in the earliest stages of the disease. Rather, an increased width of the distribution of T2 (T2 heterogeneity) may better reflect subtle changes in microstructural integrity such as those present in the early stages of Alzheimer’s disease.

T2 heterogeneity as a marker of tissue integrity is a novel measure with only two known previous studies of its utility. One demonstrates that T2 heterogeneity is a useful measure in accurately determining stroke onset time in an animal model [38]. The other presented pilot data from our group, concluding that T2 heterogeneity can improve accuracy in distinguishing between healthy controls, those with MCI and Alzheimer’s disease patients, and was a more promising measure than volumetry or diffusion tensor imaging [25].

In this study, we aimed to assess the use of the width of the distribution of T2 as a marker of within-individual tissue heterogeneity and microstructural integrity. We measure T2 heterogeneity in a group of people with MCI. Studies report a variable annual conversion rate of MCI to AD (mostly ranging from 10 to 15% for studies in clinical settings [39, 40]). We hypothesised that MCI patients with the greatest T2 heterogeneity would have the greatest risk of incipient dementia and therefore experience the greatest cognitive decline over a year [39, 40]. To be clear, we expect the distribution width to increase on a patient-by-patient basis. We are not discussing heterogeneity across the MCI group, which would be explained by the variety of MCI aetiologies between individuals.

We expand previous work by describing a model of T2 dynamics through the course of Alzheimer’s disease, in comparison to healthy ageing, with a view to creating a practical biomarker which may identify neuropathology prior to significant tissue atrophy. We also report volumetry data, as this is the current standard for assessing structural change in MCI and AD.

## Methods

The analyses in this paper combine data from two prospective longitudinal studies similar in cohort demographics and study design. No participants took part in both studies. Both studies are detailed in the following section. Where data collected are not identical between cohorts, we have normalised equivalent metrics within cohort and combined data after normalisation.

### Participants

Participants fulfilling the Petersen criteria [41] for diagnosis of MCI were recruited to both studies (study 1: *n* = 30; study 2: *n* = 29). Healthy older people (HC) with no history of memory problems or significant neurological disorders were recruited as controls to each study (study 1: *n* = 61; study 2: *n* = 56). All healthy controls had Montreal Cognitive Assessment (MoCA) > 26 (study 1) or Addenbrookes Cognitive Examination 3 (ACE-III) > 88 (study 2). Seven participants originally recruited as healthy controls in study 1 were found to have MoCA scores of < 26, so they were reclassified as MCI (given the high sensitivity and specificity of the MoCA for detecting MCI at this threshold; 90% and 100%, respectively [42]). Study 1 also included 10 patients with diagnoses of Alzheimer’s disease (AD) who retained capacity to consent. AD diagnoses were made according to standard clinical criteria [43]. These sample sizes are in-line with similar studies on brain structure abnormalities in MCI and Alzheimer’s disease and are sufficient to observe significant differences in hippocampal volume. All participants underwent a battery of neuropsychological tests specific to each study, the details of which are described in supplementary information.

Subjects for both studies were recruited from local GP surgeries and memory clinics in the Bristol area (having received MCI diagnoses or reported memory problems), Join Dementia Research, Avon and Wiltshire Mental Health Partnership’s Everyone Included system, an in-house database of volunteers, replies to poster adverts or through word of mouth. All patients provided informed written consent prior to testing as according to the Declaration of Helsinki. Ethical approval was given by Frenchay NHS Research Ethics Committee.

The current analyses included all participants who had volumetry and T2 relaxometry data for both hippocampal subfields and thalamus, study 1 *n* = 90 (50 HC, 30 MCI, 10 AD), study 2 *n* = 66 (47 HC, 19 MCI). See Table 1 for demographic details (Supplementary Tables 1 and 2 show demographic, neuropsychology and MRI data for each study cohort separately).

**Table 1:** Participant demographics

	Group			
	HC	MCI	AD	Total
*N* (male to female)	97 (46:51)	49 (27:22)	10 (2:8)	156 (75:81)
Age (years)	69.3 ± 8.58	72.2 ± 9.03	77.9 ± 9.94	70.7 ± 9.05
YOE	15.8 ± 3.16	14.2 ± 2.81	13.1 ± 2.60	15.1 ± 3.13
Cognitive score (normalised to HC)	0.00 ± 1.00	− 4.08 ± 2.09	− 8.50 ± 3.15	− 1.83 ± 3.02

Data show mean ± standard deviation, combined for studies 1 and 2. Cognitive score is calculated as a *Z* score relative to the healthy control group of each study, separately, as different cognitive tests were used (study 1: MoCA; study 2: ACE-III). For this reason, the HC group by definition has a mean ± SD of 0 ± 1. *HC* healthy control, *MCI* mild cognitive impairment, *AD* Alzheimer’s disease, *YOE* years of education

A total of 20 MCI participants were followed-up after 1 year (10 from each study). Cognitive function was tested at baseline and follow-up using the MoCA in study 1 and the ACE-III in study 2. We assessed cognitive decline as an overall change in this cognitive test score over the year follow-up period. Although some MCI patients may have converted to dementia over the year, conversion to dementia was never a formal outcome of this study. The reason for this is that we recruited from a range of sites with highly variable clinical follow-up periods for MCI patients—indeed, some sites discharge MCI patients without a planned follow-up. This led to our decision to use our own measure of cognitive change as the outcome of interest in this study.

### Imaging parameters

Scans for both studies were acquired at CRICBristol, University of Bristol, UK, on the same Siemens Magnetom Skyra 3T system equipped with a parallel transmit body coil and a 32-channel head receiver array coil. The two studies used similar, but slightly different scanning protocols.

#### Study 1

This protocol has been previously described by Knight et al. [25]. The imaging protocol included a 3D T1-weighted whole-brain magnetization prepared rapid acquisition gradient-echo (MPRAGE) and 2D multi-contrast multi-spin-echo (CPMG).

MPRAGE: Coronal, whole-brain, repetition time (TR) 2200 ms, echo time (TE) 2.42 ms, inversion time (TI) 900 ms, flip angle 9°, acquired resolution 0.68 × 0.68 × 1.60 mm, acquired matrix size 152 × 320 × 144, reconstructed resolution 0.34 × 0.34 × 1.60 mm (after twofold interpolation in-plane by zero-filling in *k*-space), reconstructed matrix size 540 × 640 × 144, GRAPPA factor 2. Acquisition time: 5:25 min.

CPMG: Coronal, TR 4500 ms, TE 12 ms, number of echoes 10, echo spacing 12 ms, acquired resolution 0.68 × 0.68 × 1.7 mm inclusive of 15% slice gap, acquired matrix size 152 × 320, 34 slices, interleaved slice order, reconstructed resolution 0.34 × 0.34 × 1.7 mm (after twofold interpolation in-plane by zero-filling in *k*-space, and inclusive of 15% slice gap), reconstructed matrix size 540 × 640, 34 slices, GRAPPA factor 2. Acquisition time: 11:07 min.

#### Study 2

The imaging protocol included a 3D T1-weighted whole-brain MPRAGE and 2D multi-contrast turbo spin-echo (TSE).

MPRAGE: Sagittal, whole-brain, TR 2200 ms, TE 2.28 ms, TI 900 ms, flip angle 9°, FOV 220 × 220 × 179 mm, acquired resolution 0.86 × 0.86 × 0.86 mm, acquired matrix size 256 × 256 × 208. Acquisition time: 5:07 min.

Multi-contrast TSE: Coronal, TR 7500 ms, number of echoes: 3, TE 9.1, 72 and 136 ms, acquired resolution 0.69 × 0.69 × 1.5 mm, reconstructed resolution 0.34 × 0.34 × 1.5 mm (after 2-fold interpolation in-plane by zero-filling in *k*-space, and inclusive of 15% slice gap), GRAPPA factor 2, FOV 220 × 220 × 34, acquired matrix size 270 × 320 × 58. Acquisition time: 5:09 min.

CPMG and TSE scans were not ‘whole-brain’, their coverage only extending approx. 1 cm beyond anterior and posterior ends of the hippocampus. These scans were tilted such that the hippocampal body lay perpendicular to the slice acquisition plane. These scans also included the entirety of thalamus.

The two distinct methods of measuring T2 (CPMG vs TSE) will give inherently different values for T2 midpoint and heterogeneity between studies (see supplementary information). Relationships to variables such as age and cognitive score should be similar, given they are sensitive to the same tissue properties.

### Imaging analyses

All analyses were performed at CRICBristol in a Linux cluster environment. All analyses were carried out in single-subject native space.

CPMG and TSE scans were brain-extracted using FSL’s *bet2* on the first echo in the series [44]. All extracted images were visually inspected for quality and rerun with different fractional intensity thresholds or gradient parameters where necessary. Fractional intensity threshold was typically set between 0.2–0.3. MPRAGE images were brain-extracted using *vbm8bet* (in-house script) and bias-field-corrected using FSL FAST [45]. T2 maps were created in MATLAB from multi-echo sequences by fitting logarithmic-space mono-exponential decay functions to each voxel series (overall summary of T2 calculation is shown in Knight et al. [25]). The first echo of CPMG was always excluded. A sum-of-echoes image was created in order to have one structural image representing the entire multi-echo sequence. This image was used for segmentation.

Hippocampus was automatically masked using the Automatic Segmentation of Hippocampal Subfields (ASHS) software package [46] (version: rev103, dated 12 June 2014; UPENN memory centre atlas dated 16 April 2014). CA1, CA2, CA3, dentate gyrus, subiculum and miscellaneous were combined to form a total hippocampus mask. This was overlaid onto T2 maps, giving a value of T2 for each voxel of hippocampus.

Whole thalamus masks were created using Freesurfer v6.0, using MPRAGE scans as input images [47]. After extraction from the Freesurfer segmentation image and registration to T2-space (TSE or CPMG) using FSL’s FLIRT, thalamus masks were then overlaid onto T2 maps, and descriptive statistics were calculated, similarly to hippocampus. These automated masking programmes have demonstrated high accuracy whilst minimising subjective rater bias, without the need for group blinding.

### Modelling T2 heterogeneity

Distribution histograms were capped at 30 ms and 200 ms, as values outside these regions are unphysiological in brain tissue at 3T. The free-to-download MATLAB function ‘fitmethis’ [48] was used to fit 18 different distribution functions (see supplementary information) to left and right hemisphere ROIs individually, using maximum likelihood estimation. Akaike Information Criteria (AIC) was calculated for each distribution type. The best fitting model was determined by the lowest AIC. The most frequent best-fitting model was recorded for the hippocampus and thalamus, and subsequent statistics calculated therefrom.

### Statistical analysis

ICV-corrected volumes, T2 metrics and cognitive scores were converted into *Z* scores for each study separately and pooled, with healthy controls of each study as a reference population. MANCOVA results comparing the two cohorts before and after this normalisation can be found in Supplementary Tables 3 and 4. Model parameters of T2 distributions were compared between groups using ANCOVA, with age as a covariate. Years of education and study (1 or 2) were included as the covariates in all models but did not significantly contribute to the model in any case. We also ran models using gender as a covariate, the results of which are shown in supplementary information (Supplementary Table 5), but the overall pattern of results was unchanged. Reported models correct for age but not years of education, study, or gender. Homogeneity of variances was tested using Levene’s test, which was not significant for any test. Graphs show estimated marginal means from this analysis. Post-hoc pairwise comparisons were carried out using sidak correction for multiple comparisons (corrected *p* values are shown as ‘*p*_sidak_’). Ability of volume and T2 to predict cognitive decline was assessed using linear regression, with follow-up cognition as the dependent variable and baseline cognition and age as covariates:

$$ \mathrm{Follow}-\mathrm{up}\ \mathrm{Cognition}={\beta}_{\mathrm{intercept}}+{\beta}_{\mathrm{age}}\left(\mathrm{Age}\right)+{\beta}_{\mathrm{BLCog}}\left(\mathrm{BaselineCognition}\right)+{\beta}_{\mathrm{Volume}/\mathrm{T}2}\left(\mathrm{Volume}/\mathrm{T}2\right)+\mathrm{error} $$

Gender and study were also explored as covariates in these linear regression models; however, in no model were they significant predictors. *Z* scores for the latter analysis were calculated relative to each study’s MCI population only.

Linear regressions were used to assess the strength of the relationship between age and T2 statistics in healthy controls. All reported *p* values are two-tailed. Balance tests were not carried out on demographic for reasons detailed by Mutz et al. [49].

Data handling and storage was carried out using MathWorks MATLAB 2015a (with statistics and machine learning toolbox) and Microsoft Excel 2016. Statistical analysis was performed in IBM SPSS Statistics 24. Graphs were produced using GraphPad Prism v7.

## Results

Demographic details for the entire cohort can be found in Table 1. Separated demographic information for study 1 and study 2 can be found in Supplementary Tables 1 and 2, respectively, including specific cognitive test scores for each group.

### Model fitting to describe T2 distribution characteristics

**Fig. 1:**
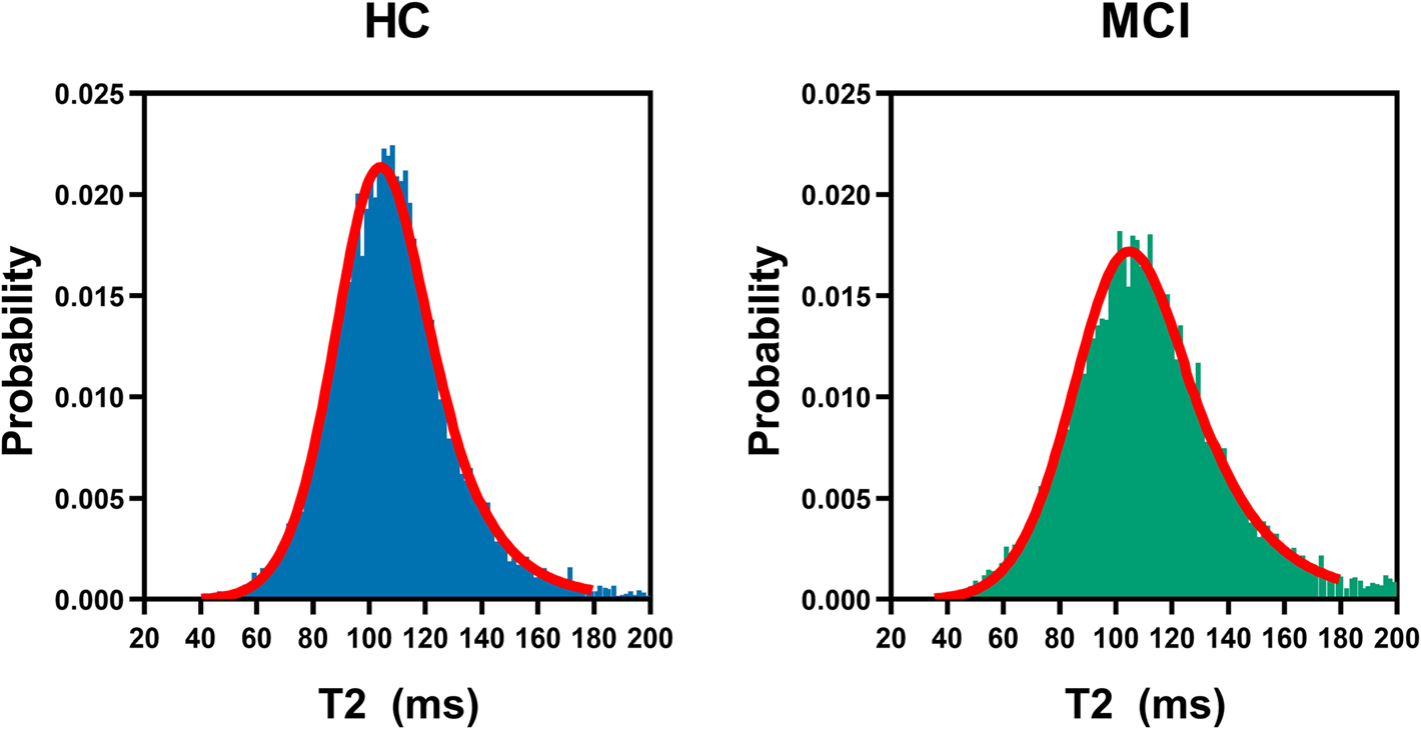
Hippocampal T2 relaxation time histograms for example participants. Left: Healthy control, 69-year-old female (*μ* = 4.68, *σ* = .112). Right: MCI, 87-year-old male (*μ* = 4.71; *σ* = .135). Left hippocampus is shown in both examples. Red lines on each graph represent log-logistic distribution curves fitted to each participant’s data

T2 distributions in the hippocampus and thalamus (Fig. 1) were best described in the majority of cases by a log-logistic distribution function (as determined by the lowest AIC). Log-logistic distribution is defined as:

$$ f\ \left(x|\mu, \sigma \right)=\frac{1}{\sigma}\frac{1}{x}\frac{\exp \left(\mathrm{z}\right)}{{\left[1+\exp \left(\mathrm{z}\right)\right]}^2},\mathrm{where}\ z=\frac{\log (x)-\mu }{\sigma } $$

where *μ* and *σ* denote the log-median value (midpoint) and distribution shape (heterogeneity), respectively. Values for hippocampus and thalamus volume and T2 model parameters can be found in Supplementary Tables 3 and 4.

### T2 heterogeneity, but not midpoint, differentiates healthy older adults from those with MCI

#### T2 midpoint (μ)

**Fig. 2:**
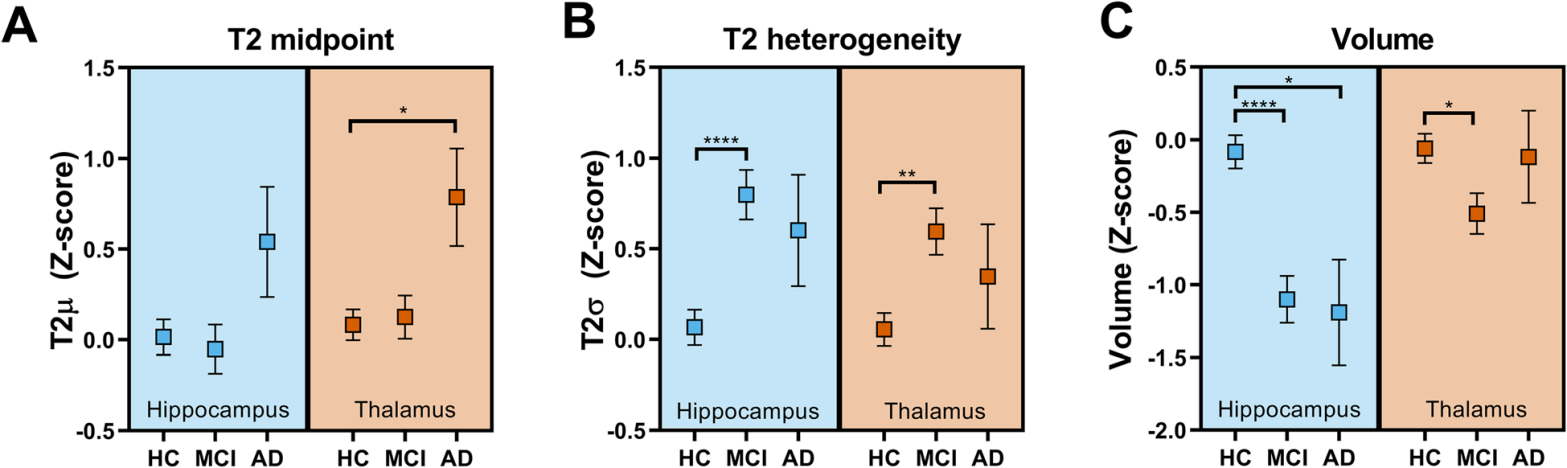
Group comparisons for structural measures in hippocampus and thalamus. Comparisons are shown for **a** T2 midpoint (*μ*), **b** T2 heterogeneity (*σ*) and **c** ICV-corrected volume. Values shown are estimated marginal means after correcting for the effect of age. Error bars show marginal means ± standard error. Asterisks represent Sidak pairwise comparisons *p* values (*p < .05; **p < .01; ****p < .0001)

There was no significant difference between HC, MCI and AD groups (*F*(2, 152) = 1.61, *p* = .204; Fig. 2a) on T2 midpoint in the hippocampus. Although T2 midpoint was higher in the AD group than other groups, this effect was not statistically significant compared to either healthy controls (*p*_Sidak_ = .283) or the MCI group (*p*_Sidak_ = .211). There was no significant difference between healthy control and MCI groups (*p*_Sidak_ = .971).

We found a significant effect of group on T2 midpoint in the thalamus (*F*(2, 152) = 3.10, *p* = .048; Fig. 2a). Post hoc analyses revealed that this was driven by an increase in T2 in the AD group (HC vs AD: *p*_Sidak_ = .042; MCI vs AD: *p*_Sidak_ = .073). As in the hippocampus, there was no significant difference between healthy controls and the MCI group (*p*_Sidak_ = .989).

#### T2 heterogeneity (*σ*)

There was a significant effect of group on hippocampal T2 heterogeneity (*F*(2, 152) = 9.76, *p* = .0001; Fig. 2b). Pairwise comparisons revealed a significantly wider distribution in the MCI group compared to healthy controls (*p*_Sidak_ < .0001). There was no significant further change from MCI to AD (*p*_Sidak_ = .913), nor was there a significant difference between healthy controls and the AD group (*p*_Sidak_ = .273).

There was a significant difference in thalamic T2σ between groups (*F*(2, 152) = 5.90, *p* = .003; Fig. 2b). Post hoc pairwise comparisons revealed a significantly increased T2σ in the MCI group compared to HCs (*p*_Sidak_ = .002). In line with findings in the hippocampus, we observed no significant difference between MCI and AD groups (*p*_Sidak_ = .813) or between healthy controls and the AD group (*p*_Sidak_ = .710).

#### Volume

We found a significant effect of group on volume (*F*(2, 152) = 14.8, *p* < .0001; Fig. 2c). Pairwise comparisons revealed a significantly smaller volume in the MCI group compared to healthy controls (*p*_Sidak_ < .0001), as well as a significant difference between HC and AD groups (*p*_Sidak_ = .014). There was no significant further increase from MCI to AD (*p*_Sidak_ = .994).

There was a significant difference in thalamic volume between groups (*F*(2, 152) = 3.41, *p* = .036; Fig. 2c). Post hoc pairwise comparisons revealed a significantly smaller volume in the MCI group compared to HCs (*p*_Sidak_ = .032).

### T2 heterogeneity predicts cognitive decline in mild cognitive impairment

**Fig. 3:**
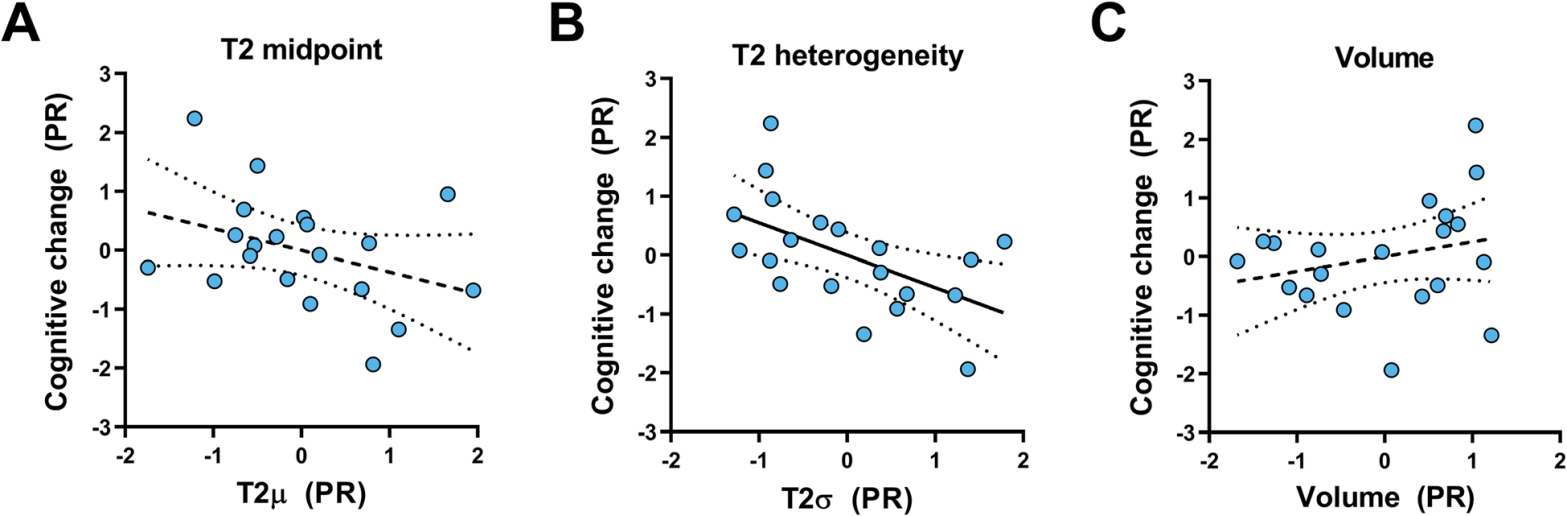
The ability of hippocampal metrics to predict cognitive change over 1 year. Data shown are partial residual (PR) plots for hippocampal structural measures predicting follow-up cognitive score, correcting for age and baseline cognition. *Y*-axes show standardised residuals from linear regression of age and baseline cognitive score predicting follow-up cognitive score. *X*-axes also show standardised residuals with the same predictors, predicting hippocampal T2 midpoint (**a**), T2 heterogeneity (**b**) or ICV-corrected volume (**c**). Solid black lines represent linear regression slopes with *p* < .05. Dotted lines represent those with *p* > .05. Regression lines are shown with ± 95% confidence intervals

Hippocampal T2 heterogeneity significantly predicted follow-up cognitive score, after accounting for baseline cognitive score and age (*R*^2^ = .387, *F*(3, 16)=3.37, *p* = .045; Fig. 3b). T2 heterogeneity was the sole significant individual predictor in this model (*β*_T2σ_ = −.601, *p*_T2σ_ = .018). Cognitive change over time was not predicted by this method by either hippocampal T2μ (*R*^2^ = .241, *F*(3, 16)=1.69, *p* = .209; *β*_T2μ_ = −.377, *p*_T2μ_ = .132; Fig. 3a) or hippocampal volume (*R*^2^ = .177, *F*(3, 16)=1.15, *p* = .361; *β*_vol_ = .263, *p*_vol_ = .315; Fig. 3c).

### Effects of age on T2 relaxometry and volume in individuals with normal cognition

#### T2 midpoint (μ)

**Fig. 4:**
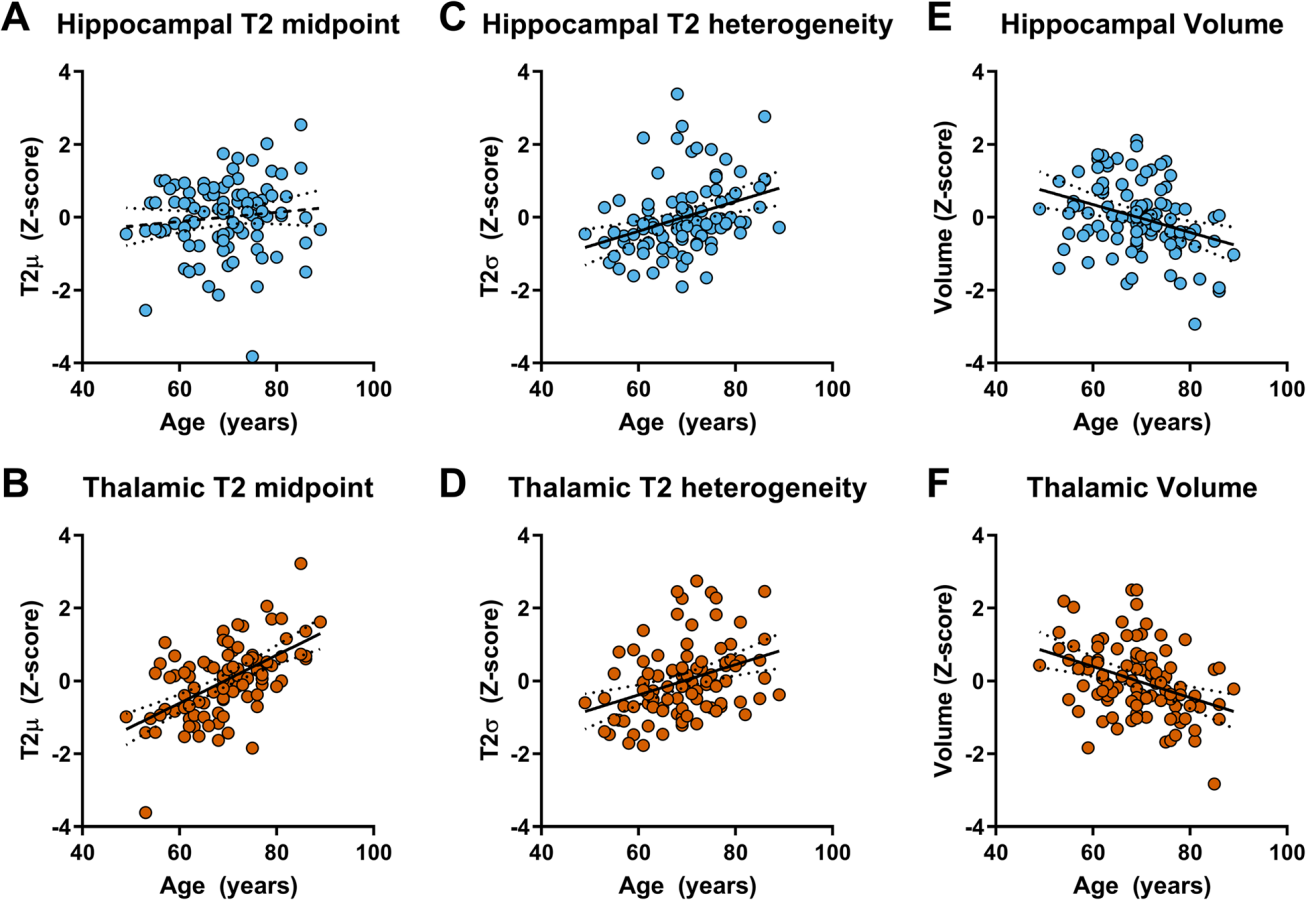
Linear regressions for age predicting T2 model descriptive parameters in hippocampus and thalamus. Regressions shown are between age and hippocampal T2μ (**a**), thalamic T2μ (**b**), hippocampal T2σ (**c**), thalamic T2σ (**d**), hippocampal volume (**e**) and thalamic volume (**f**). All volumes were normalised to ICV. Solid black lines represent linear regression slopes with *p* < .05. Dashed lines represent those with *p* > .05. Regression lines are shown with ± 95% confidence intervals

There was no statistically significant relationship between age and hippocampal T2 midpoint (T2μ, *R*^2^ = .012, *p* = .289, *n* = 97; Fig. 4a) in cognitively normal individuals. In the thalamus, age was a strong positive predictor of T2μ (*R*^2^ = .320, *p* < .0001, *n* = 97; Fig. 4b).

#### T2 heterogeneity (*σ*)

Age was a significant positive predictor of T2 heterogeneity in the hippocampus (T2σ, *R*^2^ = .122, *p* = .0004; Fig. 4c) and thalamus (*R*^2^ = .127, *p* = .0003; Fig. 4d) in cognitively normal individuals.

#### Volume

Age was a significant positive predictor of volume in the hippocampus (*R*^2^ = .106, *p* = .001; Fig. 4e) and thalamus (*R*^2^ = .134, *p* = .0002; Fig. 4e) in cognitively normal individuals.

## Discussion

We show that the width of the distribution of T2 in the hippocampus and the thalamus differentiates healthy older adults from those with mild cognitive impairment, while the T2 midpoint does not. Heterogeneity of T2 may therefore be a marker of structural integrity, which has potential detect early signs of Alzheimer’s disease pathology. Although ageing affects T2, even after controlling for age, T2 heterogeneity predicted decline whereas hippocampal volume and T2 midpoint did not.

**Fig. 5:**
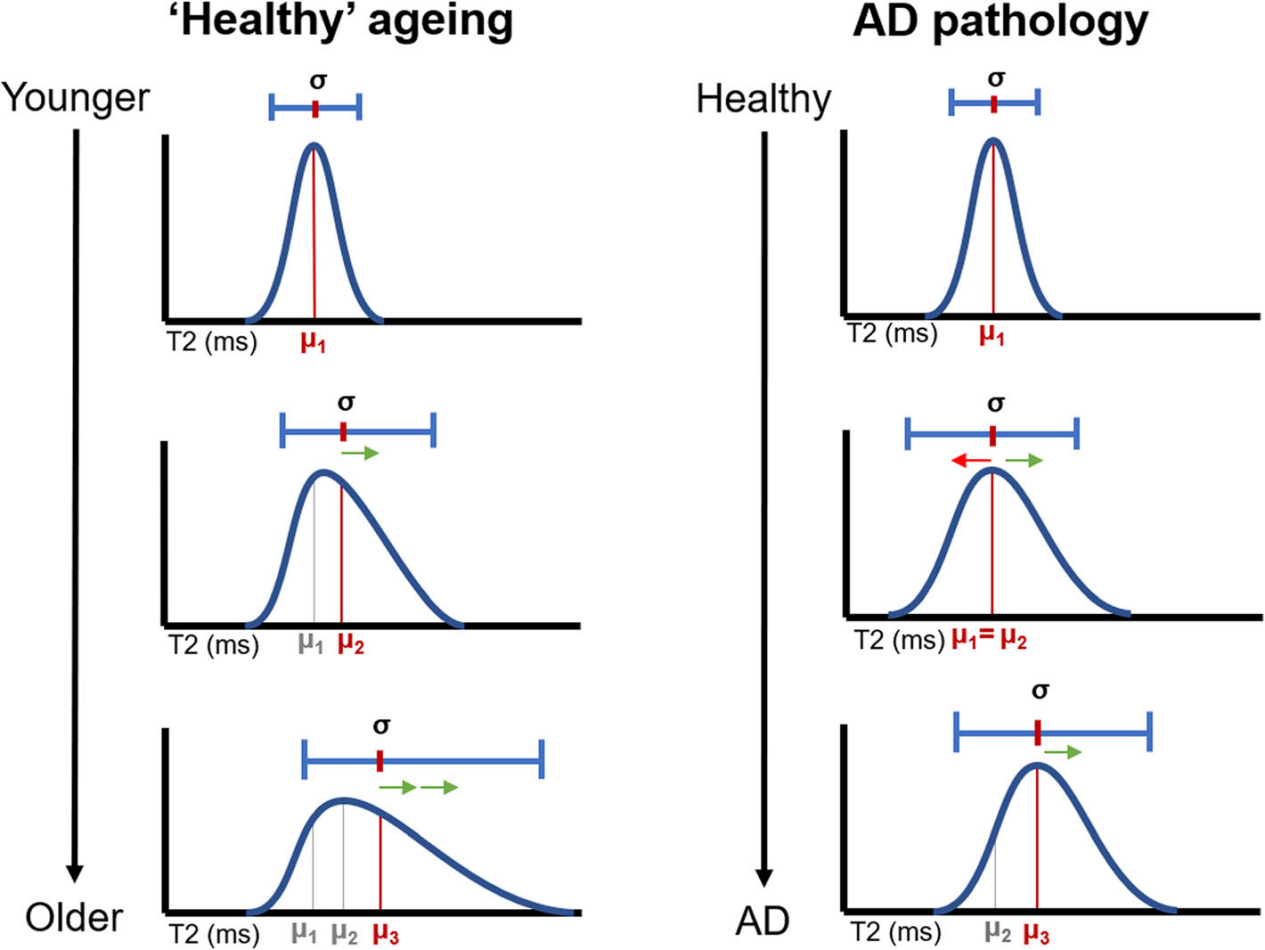
Schematic diagram of T2 distribution profiles in ageing and Alzheimer’s disease. Midpoint values for each hypothetical distribution are represented by orange bars and ‘*μ*’ markers on each *x*-axis. Green and red arrows represent factors that increase or decrease T2, respectively. The number of arrows represents the relative dominance of each effect. In summary, the model suggests that factors that increase T2 are present in both healthy ageing and Alzheimer’s pathology; however, factors that decrease T2 are more dominant in Alzheimer’s disease. Early Alzheimer’s disease pathology is characterised by an increase the distribution without an increase in the midpoint

Based on the presented T2 relaxometry data, we propose the following model in Fig. 5 where healthy ageing is characterised by a relative dominance of factors that increase T2 over factors that decrease T2, particularly in the thalamus. Incipient Alzheimer’s disease may be characterised by additional factors that decrease T2, balancing out the effects of T2-increasing factors on T2 midpoint to some extent. This leads to an increasing width of the distribution of T2 without necessarily changing the midpoint in prodromal AD. We see this in our MCI cohort, who are at increased risk of a later diagnosis of AD [39, 40, 50]. In later stages of disease, after a diagnosis of Alzheimer’s disease, factors that increase T2 may predominate. This model explains these data and ties together previous seemingly conflicting literature such as the discrepancy between human and animal literature of T2 changes due to Alzheimer’s disease (see supplementary information for full discussion on this point).

### T2-prolonging factors are dominant in healthy ageing and later stage Alzheimer’s disease

The primary causes of variability in T2 are content and mobility of water. T2 increases as water mobility increases [51]. The amount of free water in a region can be partially attributed to the inverse of the compartmentalisation of the water, as is caused by cell membrane disruption. As cells die, whether due to normal ageing processes [16, 17] or pathology, cell membranes become damaged, thereby increasing the amount of free water within a tissue [52]. The breakdown of myelinated structures also causes T2 to increase in white matter and can be caused by both ageing and Alzheimer’s disease [24, 53–57], as well as other conditions including vascular dementia [58]. This leads to an increase in T2 both in healthy ageing and in Alzheimer’s disease, even in early stages, as a result of microstructural damage. In support of this, this study shows a significantly longer T2 in the thalamus of Alzheimer’s disease patients compared to healthy controls, as well as in cognitively normal older people.

Additionally, we show that T2 heterogeneity is predicted by age in both the thalamus and hippocampus, an effect that would be expected from uneven increases in T2 across the region. However, T2 does not appear to increase significantly in the hippocampus either with age or disease progression, except perhaps at later stages of the disease course, after a diagnosis of Alzheimer’s disease. One explanation is that the increase in T2 is balanced out in the hippocampus by T2-shortening factors that are present even prior to MCI diagnosis. This is discussed further in the following sections.

### T2-shortening factors may indicate pathology beyond the effects of ageing

Dense protein structures (e.g. Aβ, NFTs) and paramagnetic materials (e.g. iron) cause T2 to decrease due to an increased macromolecule-to-water ratio and the restriction of water motility in the extracellular space. Neuropathology defined by overexpression of such factors might therefore be expected to decrease T2 in localised regions of deposition. In the case of Alzheimer’s pathology, this would occur in the hippocampus and thalamus, balancing out the T2-prolonging factors discussed previously. In support of this, this study shows a substantial increase in distribution width of T2 in both hippocampus and thalamus, after correcting for age, in people with MCI compared to healthy controls. Furthermore, the study also shows no increase of T2 midpoint in MCI compared to controls, a result to be expected given counteracting factors increasing and decreasing T2.

**Fig. 6:**
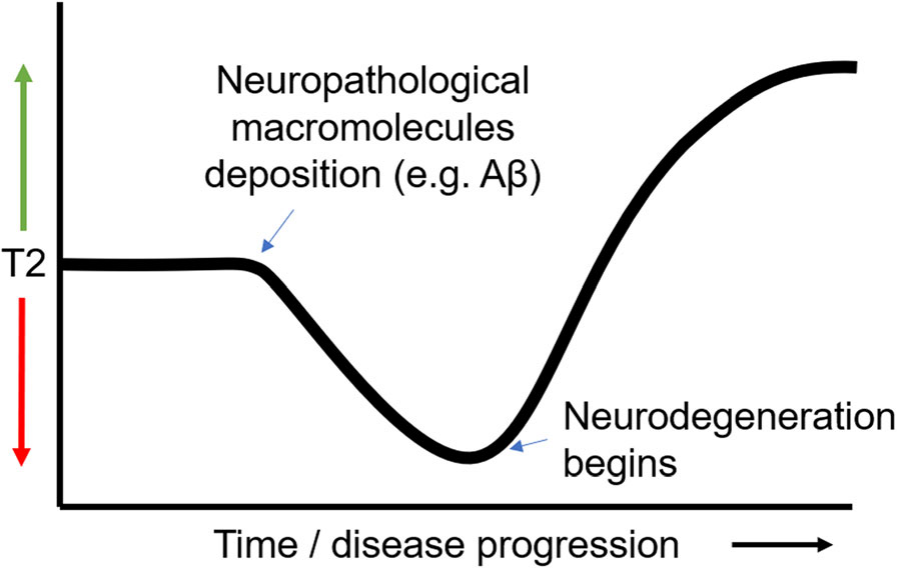
Theoretical model of T2 dynamics in a single voxel in the brain throughout the course of Alzheimer’s disease. A given region in the brain of someone with incipient Alzheimer’s disease would consist of many voxels at different stages of this curve, depending on the degree of Alzheimer’s pathology in a given location. This heterogeneity is what will cause the average or midpoint T2 to remain relatively static, and the distribution width to increase, until very late stages when all voxels reach the ‘high T2’ state

The described model is further supported by a previous study by Su et al. [59]. The cross-sectional results of their study revealed that Alzheimer’s disease patients had significantly reduced T2 compared to healthy controls. However, longitudinally, T2 in Alzheimer’s disease patients was seen to increase. The currently presented model explains these results in terms of a shift in the dominance of factors that increase or decrease T2 throughout the progression of Alzheimer’s disease. Macromolecular pathological hallmarks cause T2 to decrease in the first instance, which later causes physical damage to the structure, causing T2 to increase as the disease progresses (Fig. 6), as is seen in the majority of studies on T2 in Alzheimer’s disease [26–30].

There is, of course, considerable debate as to the role of plaques in Alzheimer’s disease pathology (for a review see [60]) and some question as to the disease-specificity of iron accumulation [36, 61]. Indeed, plaques have been found in the brains of many people without any other sign of Alzheimer’s disease, particularly in the hippocampus [62, 63]. Oligomeric Aβ, however, could still have T2-shortening effects in the brains of people with early Alzheimer’s disease. The presence of some T2-shortening factors even in those with no Alzheimer’s disease-specific pathology could explain the lack of correlation between hippocampal T2 midpoint and age in healthy control participants. In further support of this, we do see a strong correlation between T2 midpoint and age in the thalamus, a region which is less likely to display pathology in a healthy control cohort [14, 63]. This is discussed further in supplementary information. T2-shortening could also be caused by iron in microglia which are recruited in response to inflammation. Although inflammation is a factor in Alzheimer’s disease, it could also be present in response to comorbidities like cardiovascular disease [64]. Conversely, cardiovascular disease could also reduce blood flow to the brain, potentially reducing the iron and causing T2 to increase. Unfortunately, amyloid and iron status of the current studies’ participants were not available, so the higher number of voxels with low T2 cannot be directly attributed to either factor. Future work could aim to colocalise areas of low T2 with Aβ, for example with positron emission tomography (PET), and brain iron levels by measuring field dependent relaxation rate increase (FDRI) as was conducted by Raven et al. [65].

Despite some presence of Aβ and iron in healthy ageing, various studies suggest that the two factors combine in Alzheimer’s pathology, leading to the much greater T2-shortening effects seen in Alzheimer’s disease. A study by El Tannir El Tayara et al. [23] showed that T2 in the hippocampus (specifically in the subiculum) was decreased in a mouse model of Alzheimer’s disease that produced amyloid deposits (APP/PS1), compared to another model that does not form such deposits (PS1). The authors attribute this, at least in part, to the colocalization of amyloid and iron. Such histological colocalization has also been reported by Falangola et al. [66]. Excess iron can not only contribute to oxidative stress in and of itself, but can also contribute to Aβ and NFT misfolding [67], thereby exacerbating Alzheimer’s pathology. Numerous studies are supportive of the idea that a combination of iron and Aβ cause significant T2 shortening [23, 35, 66, 68–73]. This also has implications for Lewy body diseases such as Parkinson’s disease, which is also characterised by increased iron deposition [36].

### Potential clinical utility of T2 relaxometry

Understanding T2 dynamics in preclinical Alzheimer’s disease and healthy ageing offers the potential for great clinical benefit. If Alzheimer’s pathology can be detected using MRI prior to the onset of hippocampal atrophy, significant change in cognition, or loss of daily independence, patients may receive treatment much earlier—at a stage where neurodegenerative damage is preventable or even reversible. Additionally, as our pilot data show [25], T2 heterogeneity outperforms more traditional measures of microstructural integrity in the identifying pathology.

In this study, we show that T2 heterogeneity can predict cognitive decline in the MCI group where volume and T2 midpoint cannot. This effect was significant after regressing out the effect of age suggesting that it does relate to pathology or other age-independent brain changes. Furthermore, any test-retest variability in the cognitive tests used would likely only introduce noise rather than systematic bias. We therefore believe this result to be robust.

Hippocampal volume is the one of the most widely studied and effective predictors of cognitive decline (for a review, see de Flores et al. [74]). However, rather than measuring pathology itself, volumetry measures tissue atrophy, a consequence of pathology. T2 increases also measure consequences of pathology, in the form of increased regional CSF, oedema or cell membrane breakdown; however, it is a more sensitive measure and may indicate subtle damage before macroscopic atrophy is detectable. Furthermore, T2 decreases may measure key features of Alzheimer’s pathology itself, such as iron, Aβ and NFT deposition that can occur before hippocampal shrinkage [75]. Measuring T2 heterogeneity allows these opposing factors to be considered, as they may indicate slightly damaged tissue that has the potential for therapeutic rescue. Measuring T2 distribution width compared to age-corrected normative data may be indicative of physical damage beyond what should be expected for a given age. Given systematic differences in T2 between pulse sequences (as seen in Supplementary Tables 1–4), exact normative data would have to be standardised for a given sequence. However, as we see consistent results across two cohorts with two different pulse sequences, we expect these results to be highly generalisable across sequences. These markers may compliment or even surpass volumetry in predicting future cognitive decline. As neuroimaging, often MRI, is part of routine clinical screening processes for neurological disease, this method is highly practical and easily translatable.

It is important to highlight that even though our results are largely discussed in the context of AD, an increase in the distribution of T2 is likely not specific to AD per se, but rather may be a highly versatile novel measure of microstructural integrity that can be applied to the diagnosis of many diseases. It is likely to be particularly useful in any disease characterised by factors which both increase and decrease T2, in which changes in T2 midpoint would be masked. This may include many neurodegenerative disorders, particularly those where age is a risk factor, such as dementia with Lewy bodies, Parkinson’s disease or vascular dementia. MCI is also a risk factor for these disorders [76, 77], and pathology for these conditions is likely present within our MCI population. As with any structural measure, it will be the spatial and temporal patterns of microstructural changes throughout the brain which may be specific to a given disease. This study focused on the MTL and thalamus in groups with high risk of AD pathology, thus discussion centres mostly around AD. However, non-AD disease pathology may also be present in our MCI cohort also causing increased T2 heterogeneity in the hippocampus and/or thalamus.

With further research to characterise the pattern of microstructural changes in T2 distribution and volume within the brain, perhaps by looking in closer detail at subfields of the MTL, T2 heterogeneity may be used to develop more specific diagnostic criteria early on in the disease process. If this is the case, MRI may become a non-invasive alternative to CSF biomarker analysis and a cheaper option than amyloid or tau PET scanning, both of which can detect very early AD pathology [78, 79]. Furthermore, although CSF biomarkers provide a good overview of the presence of pathology, T2 heterogeneity allows direct quantification of tissue which, although damaged, may stand a chance of therapeutic rescue, and may therefore predict treatment efficacy on a patient by patient basis. The combined value of T2 heterogeneity and CSF biomarkers is, of course, an exciting avenue for future research.

In addition to the clinical utility of T2 heterogeneity described here, T2 heterogeneity also has potential for use in basic and translational research. Current studies of the function of human hippocampus and its constituent subfields, for example, often involve assessing relationships with volume, despite limitations of the ‘bigger is better’ hypothesis (see review by Petten [80]). T2 heterogeneity may be used to identify tissue that is extant but dysfunctional, which may otherwise confound volumetry, leading to more accurate assessment of the amount of ‘healthy’ tissue present. This, of course, has the potential for application to other brain areas and may contribute to an overall better understanding of brain-behaviour relationships in health and disease.

### Limitations

With the exception of some of those who have actually received a clinical diagnosis of Alzheimer’s disease in study 1, the amyloid status of these participants is unknown. Amyloid (measured either in CSF or using PET) is one of the most commonly used biomarkers to increase certainty of the presence of Alzheimer’s disease pathology. Those who present with mild cognitive impairment often are only classified as MCI based on presentation of cognitive symptoms. Such cognitive impairment could be caused by factors other than Alzheimer’s pathology, including other dementias, stroke, pharmaceutical side effects and sleep problems to name a few. Further work is required to understand the ability of T2 heterogeneity to rule out causes of MCI not related to dementia.

Secondly, although we present results in a relatively large sample of healthy older controls and people with MCI, we are limited by our small sample of Alzheimer’s disease patients. This is primarily because they were only recruited as a part of study 1. This limits the statistical significance of some of the effects that we describe, and therefore conclusions from this group are slightly tentative. This is acknowledged throughout interpretation of these results, which we expect to be reproducible with a larger sample size. The lack of any observed statistical difference between MCI and AD groups is also further discussed in supplementary information.

Thirdly, this study combines two distinct participant cohorts, the methodology of which differ in two key ways: (i) the test used to measure general cognitive ability (study 1: MoCA; study 2: ACE-III) and (ii) the MRI sequence used to quantitatively assess T2 (study 1: 10-echo CPMG; study 2: 3-echo TSE). For these data, we have normalised within-cohort (calculated *Z* scores) and combined data after normalisation. Given that the cohorts are similar in almost every other way, and these methods are purported to measure the same underlying principles, the benefits of a larger sample size provide ample justification for combining cohorts as we have done.

Finally, the only regions studied here, hippocampus and thalamus, are both regions known to be affected by Alzheimer’s pathology at early stages. Future studies would benefit from exploring T2 dynamics in other brain regions, including those that are not directly implicated in early Alzheimer’s disease. This is not possible with existing data for either study 1 or study 2, as the multi-echo T2 scans acquired do not cover the whole brain. Future analyses should also focus on subdivisions in these regions, such as T2 differences between MTL subfields and across individual thalamic nuclei, which have different susceptibility to AD pathology.

## Conclusions

In this paper, we show that T2 heterogeneity is a good measure of microstructural integrity of brain tissue. We propose a model (Fig. 5) that suggests factors that increase T2 are indicative of microstructural damage but are not necessarily specific signs of Alzheimer’s pathology. Rather, factors that decrease T2 are prevalent in Alzheimer’s pathology and may occur in the earliest stages of disease (Fig. 6). These two opposing forces act to balance out the mean in prodromal Alzheimer’s disease, causing varied results in the human literature. The model makes specific and testable predictions about the temporal dynamics of T2 alterations throughout ageing and prodromal Alzheimer’s disease. It also highlights potential early indicators of Alzheimer’s disease, allowing Alzheimer’s disease-related cognitive decline to be distinguished from that seen in healthy ageing. We show that T2 heterogeneity surpasses midpoint T2 and the more established measure of volumetry in predicting cognitive decline in those with MCI.

This study represents one of the first studies of T2 heterogeneity within the brain in MCI and Alzheimer’s disease, and the first to show its utility in predicting cognitive decline.

## Supplementary information

**Additional file 1: Supplementary information**. **Supplementary Table 1**. Study 1 cohort information. **Supplementary Table 2**. Study 2 cohort information. **Supplementary Table 3**. Multivariate ANOVA results for testing between-study differences in raw volume and T2 data. **Supplementary Table 4**. Multivariate ANOVA results for testing between-study differences in volume and T2 data after being normalised to each study’s healthy control group. **Supplementary Table 5**. ANCOVA results for predicting brain structural measures correcting for age and gender.

## Data Availability

The datasets used during the current study are available from the corresponding author on reasonable request.
