# Supplementary material for: T2 heterogeneity: a novel marker of microstructural integrity associated with cognitive decline in people with mild cognitive impairment
Source: Alzheimers Res Ther. 2020 Sep 10;12:105. doi: 10.1186/s13195-020-00672-9 (PMC7488446; doi:10.1186/s13195-020-00672-9)
Supplement: Supplementary file 1 — Additional file 1: Supplementary information. Supplementary Table 1. Study 1 cohort information. Supplementary Table 2. Study 2 cohort information. Supplementary Table 3. Multivariate ANOVA results for testing between-study differences in raw volume and T2 data. Supplementary Table 4. Multivariate ANOVA results for testing between-study differences in volume and T2 data after being normalised to each study’s healthy control group. Supplementary Table 5. ANCOVA results for predicting brain structural measures correcting for age and gender. [file 13195_2020_672_MOESM1_ESM.docx]

Supplementary information

### Details of neuropsychological testing battery

Participants in both studies conducted a variety of cognitive tests to assess various aspects of memory and cognition. These tests are detailed below.

#### Study 1:

Montreal Cognitive Assessment (MoCA): One of the most widely used brief cognitive rating tools, the MoCA is a 30-point neuropsychological evaluation tool that has been shown to accurately reflect the presence of cognitive impairment (1). It takes approximately 10 minutes to administer, and assesses attention, memory, language, fluency and visuospatial cognitive domains. A score of 26 or below is thought to be a likely indicator of cognitive impairment.

Hopkins Verbal Learning Task-Revised (HVLT-R): A brief clinically-used cognitive assessment tool designed to quickly assess verbal memory (2). A list of 12 words, in 3 semantic categories are read aloud to the subject. Immediately following this, free recall of the list is tested. This is repeated twice more, and delayed recall is tested after 20 minutes.

Brief Visuospatial Memory Test-Revised (BVMT-R): A brief clinically-used cognitive assessment tool designed to quickly assess visual memory (2). 6 ‘figures’ are shown the participant for 10 seconds. Recall of the figures is then tested, on which the participant is scored based on the accuracy and location of each figure drawing. This is repeated for 3 learning trials in total, followed by delayed recall after 20 minutes.

CANTAB Paired Associate Learning (PAL): a computerised task of spatial memory (3). Up to 6 patterns are shown briefly to the participant in any of 6 possible locations. The task is to remember which pattern appeared in which location. Initially just 2 patterns are shown, with difficulty increasing over four trials. Shown to be highly accurate in identification of early AD (4, 5).

#### Study 2:

Addenbrookes Cognitive Examination-III (ACE-III): a well-validated collection of neuropsychological tests designed to assess various aspects of cognition, often used in clinical settings to identify signs of cognitive impairment (6). It gives scores of 5 cognitive domains: attention, memory, fluency, language, visuospatial. A total score of 100 is available. Scores below 88 are considered evidence of possible cognitive impairment. Scores below 82 indicate very high likelihood of impairment. The ACE-III has high internal consistency and possesses a Cronbach’s alpha of 0.88.

Word List: Based on the protocol of California Verbal Learning Task-II (CVLT-II). The CVLT-II is another widely used neuropsychological evaluation tool, designed to assess episodic verbal memory recall and recognition (7, 8). A list of 16 words split into 4 semantic categories (list A) was read aloud to the participant. Immediately following this, free recall was recorded. This was repeated a minimum of two times, and a maximum of 5, or until the subject reached 75% (12) correct responses. Following this learning phase, a distractor list (list B) of 16 new words, in 4 semantic categories (2 of which overlap with list A) is read, and recall recorded immediately after. Following this, free recall of list A was once again tested, followed by category-cued recall of list A. Free and cued recall of list A are tested again after 30 minutes.

Clinical Dementia Rating Scale (CDR): a 5-level rating scale designed to assess the range of severity of symptom presentation throughout the progression of dementia (9). Scores are given to each of 6 domains: memory, orientation, judgement & planning, home & hobbies, community affairs and personal care. each domain is labelled with a number depending on whether symptoms are normal (0), very mild (0.5), mild (1), moderate (2) or severe (3). Scores are obtained from a semi-structured interview with the subject, as well as a reliable informant (e.g. family member or close friend).

Rivermead Behavioural Memory Test 3 (RBMT-3): a collection of memory tests possessing high ecological validity (10, 11). RBMT-3 scores have been shown to be highly predictive of independent daily functioning, and certain subtests have been shown to be successful in aiding dementia diagnosis (12).

PAL: Also conducted in study 2 to the same protocol as in in study 1 (See above).

### Individual study cohort profiles

Demographic information, neuropsychological test data and data from MRI summaries for study 1 and study 2 can be found in Supplementary Tables 1 and 2, respectively.

Supplementary Table 1 | Study 1 cohort information.

Demographic, neuropsychology, and MRI structural measure info for study 1 cohort. Data show mean ± standard deviation. Some neuropsychological measures are missing for a small number of participants. The numbers, and which groups they belong to, is indicated in the leftmost column. HC = Healthy Control; MCI = Mild Cognitive Impairment; AD = Alzheimer’s disease; YOE = years of education; MoCA = Montreal Cognitive Assessment; HVLT-R = Hopkins Verbal Learning Task revised, BVMT = Brief Visuospatial Memory Test-Revised; PAL = CANTAB Paired Associate Learning

| **Demographics** | HC | MCI | AD | Total |
| --- | --- | --- | --- | --- |
| N (male: female) | 50 (21:29) | 30 (14:16) | 10 (2:8) | 90 (37:53) |
| Age (years) | 67.7 ± 9.03 | 70.7 ± 8.55 | 77.9 ± 9.94 | 69.8 ± 9.43 |
| YOE | 15.6 ± 2.66 | 14.5 ± 2.70 | 13.1 ± 2.60 | 14.9 ± 2.76 |
|  |  |  |  |  |
| **Neuropsychological testing** |  |  |  |  |
| MoCA (/30) | 28.0 ± 1.32 | 23.0 ± 2.83 | 16.7 ± 4.16 | 25.0 ± 4.40 |
| HVLT-R (/12) (-1 AD) |  |  |  |  |
| Trial 1 | 6.26 ± 1.69 | 4.30 ± 1.56 | 2.89 ± 1.54 | 5.26 ± 2.01 |
| Trial 2 | 9.30 ± 2.02 | 6.57 ± 1.87 | 4.56 ± 1.67 | 7.90 ± 2.56 |
| Trial 3 | 9.92 ± 2.40 | 7.07 ± 2.08 | 4.33 ± 1.50 | 8.39 ± 2.91 |
| 20m Delayed Recall | 9.52 ± 2.18 | 4.93 ± 3.14 | 0.89 ± 1.54 | 7.10 ± 3.87 |
| BVMT-R (/12)  (-2 HC, -2 MCI, -3 AD) |  |  |  |  |
| Trial 1 | 5.77 ± 2.14 | 3.29 ± 2.19 | 1.43 ± 0.53 | 4.57 ± 2.54 |
| Trial 2 | 8.15 ± 2.69 | 5.39 ± 2.75 | 1.57 ± 0.98 | 6.66 ± 3.28 |
| Trial 3 | 9.04 ± 2.65 | 5.83 ± 3.28 | 1.86 ± 1.86 | 7.39 ± 3.57 |
| 20m Delayed Recall | 8.71 ± 2.88 | 5.29 ± 3.30 | 0.71 ± 0.95 | 6.88 ± 3.80 |
| PAL Accuracy (-2 MCI, -2 AD) | .706 ± .131 | .534 ± .145 | .351 ± .079 | .618 ± .175 |
|  |  |  |  |  |
| **MRI structural measures** |  |  |  |  |
| Hippocampus |  |  |  |  |
| Volume | 1.83 ± 0.20 | 1.64 ± 0.27 | 1.51 ± 0.38 | 1.73 ± 0.27 |
| T2μ | 4.60 ± 0.03 | 4.59 ± 0.02 | 4.61 ± 0.16 | 4.60 ± 0.02 |
| T2σ | 0.10 ± 0.02 | 0.12 ± 0.02 | 0.12 ± 0.02 | 0.11 ± 0.02 |
| Thalamus |  |  |  |  |
| Volume | 4.65 ± 0.38 | 4.52 ± 0.46 | 4.50 ± 0.44 | 4.59 ± 0.42 |
| T2μ | 4.51 ± 0.03 | 4.52 ± 0.03 | 4.54 ± 0.03 | 4.59 ± 0.42 |
| T2σ | 0.13 ± 0.02 | 0.14 ± 0.01 | 0.14 ± 0.01 | 4.52 ± 0.03 |

Supplementary Table 2 | Study 2 cohort information.

Demographic, neuropsychology, and MRI structural measure info for study 1 cohort. Data show mean ± standard deviation. Some neuropsychological measures are missing for a small number of participants. The numbers, and which groups they belong to, is indicated in the leftmost column. HC = Healthy Control; MCI = Mild Cognitive Impairment; AD = Alzheimer’s disease; YOE = years of education; ACE-III = Addenbrookes Cognitive Examination-III; CDR = Clinical Dementia Rating scale; RBMT-3 = Rivermead Behavioural Memory Test version 3; PAL = CANTAB Paired Associate Learning

| **Demographics** | HC | MCI | Total |  |
| --- | --- | --- | --- | --- |
| N (male: female) | 47 (25:22) | 19 (13:6) | 66 (38:28) |  |
| Age | 71.0 ± 7.80 | 74.5 ± 9.49 | 72.0 ± 8.40 |  |
| YOE | 16.0 ± 3.63 | 13.7 ± 3.00 | 15.3 ± 3.59 |  |
|  |  |  |  |  |
| **Neuropsychological testing** |  |  |  |  |
| ACE-III (Total) /100 | 94.8 ± 3.20 | 80.2 ± 6.32 | 90.6 ± 7.92 |  |
| Attention /18 | 17.6 ± 0.64 | 15.6 ± 2.76 | 17.0 ± 1.81 |  |
| Memory /26 | 24.3 ± 2.05 | 16.5 ± 3.06 | 22.1 ± 4.26 |  |
| Fluency /14 | 12.1 ± 1.49 | 9.47 ± 2.27 | 11.3 ± 2.10 |  |
| Language /26 | 25.2 ± 1.04 | 23.8 ± 1.57 | 24.8 ± 1.35 |  |
| Visuospatial /16 | 15.6 ± 0.64 | 14.3 ± 1.59 | 15.4 ± 1.77 |  |
| Word list recall (/16) * |  |  |  |  |
| Trial 1 | 6.28 ± 2.24 | 4.05 ± 2.07 | 5.64 ± 2.40 |  |
| Trial 2 | 9.60 ± 2.33 | 6.16 ± 2.32 | 9.61 ± 2.79 |  |
| 30m Delayed Recall (-2 MCI) | 10.8 ± 2.52 | 3.64 ± 3.90 | 8.91 ± 4.32 |  |
| CDR Sum of Boxes (-6 HC, -1 MCI) | 0.20 ± 0.43 | 2.06 ± 1.79 | 0.76 ± 1.35 |  |
| RBMT-3 Scaled Score (-1 HC) | 143 ± 14.7 | 92.3 ± 25.3 | 128 ± 29.6 |  |
| PAL Accuracy (-3 HC, -4 MCI) | .685 ±.113 | .493 ± .184 | .636 ± .158 |  |
|  |  |  |  |  |
| **MRI structural measures** |  |  |  |  |
| Hippocampus |  |  |  |  |
| Volume | 1.60 ± 0.17 | 1.34 ± 0.26 | 1.53 ± 0.23 |  |
| T2μ | 4.67 ± 0.03 | 4.67 ± 0.03 | 4.67 ± 0.03 |  |
| T2σ | 0.13 ± 0.01 | 0.14 ± 0.01 | 0.13 ± 0.01 |  |
| Thalamus |  |  |  |  |
| Volume | 4.28 ± 0.34 | 4.00 ± 0.23 | 4.19 ± 0.35 |  |
| T2μ | 4.60 ± 0.03 | 4.61 ± 0.02 | 4.61 ± 0.03 |  |
| T2σ | 0.15 ± 0.01 | 0.16 ± 0.01 | 0.15 ± 0.01 |  |

### MRI statistical processing

The following distribution models were fitted to T2 histograms: Normal, exponential, gamma, logistic, t-location scale, uniform, extreme value, Rayleigh, generalized extreme value, beta, Nakagami, Rician, inverse gaussian, Birnbaum-Saunders, generalized Pareto, loglogistic, lognormal, Weibull.

Volumes are all normalised to intracranial volume (ICV) by the following formula:

$${Vol}_{norm}= \frac{{Vol}_{raw}}{ICV}\times1000$$

### Testing between-study differences in MRI parameters

To test for differences between the two studies, we ran a multivariate ANOVA for all 3 structural measures (Volume, T2μ, T2σ) in both regions (Hippocampus, Thalamus) correcting for age. The AD group was excluded to allow better study comparison. This revealed a significant effect of study on all measures, likely caused by differences in scanning sequences used (Supplementary Table 3). Critically, there was no significant interaction between study and group. After normalising data in each study to their respective healthy control group, there was no longer any effect of study on any measure, and still no interaction between study and group (Supplementary Table 4).

Supplementary Table 3 | Multivariate ANOVA results for testing between-study differences in raw volume and T2 data.

| Main Effect | DV | F(1,141) | Sig |
| --- | --- | --- | --- |
| Study | Hippocampus |  |  |
|  | Volume | 40.2 | <.0001 |
|  | T2μ | 246.5 | <.0001 |
|  | T2σ | 94.3 | <.0001 |
|  | Thalamus |  |  |
|  | Volume | 40.6 | <.0001 |
|  | T2μ | 389.0 | <.0001 |
|  | T2σ | 53.6 | <.0001 |
| Study*Group | Hippocampus |  |  |
|  | Volume | 0.97 | .326 |
|  | T2μ | 0.78 | .380 |
|  | T2σ | 0.74 | .392 |
|  | Thalamus |  |  |
|  | Volume | 1.89 | .171 |
|  | T2μ | 0.02 | .901 |
|  | T2σ | 0.70 | .403 |

Supplementary Table 4 | Multivariate ANOVA results for testing between-study differences in volume and T2 data after being normalised to each study’s healthy control group

| Main Effect | DV | F(1,141) | Sig |
| --- | --- | --- | --- |
| Study | Hippocampus |  |  |
|  | Volume | 0.52 | .473 |
|  | T2μ | 0.41 | .522 |
|  | T2σ | 0.21 | .649 |
|  | Thalamus |  |  |
|  | Volume | 0.55 | .458 |
|  | T2μ | 2.09 | .151 |
|  | T2σ | 0.37 | .544 |
| Study*Group | Hippocampus |  |  |
|  | Volume | 2.44 | .121 |
|  | T2μ | 0.78 | .380 |
|  | T2σ | 0.21 | .648 |
|  | Thalamus |  |  |
|  | Volume | 2.53 | .114 |
|  | T2μ | <0.01 | .978 |
|  | T2σ | 0.05 | .832 |

### Models correcting for Gender

We explored running all ANCOVA models comparing groups corrected for gender as well as age (Supplementary Table 5). Gender was a significant predictor of T2σ in both the hippocampus and thalamus. It was not a significant predictor for T2μ or ICV-corrected volume in either region.

Supplementary Table 5 | ANCOVA results for predicting brain structural measures correcting for age and gender.

All volumes are normalised to ICV. All structural measures are normalised to the healthy control group of their respective study, as in the main analyses.

| DV (Z-scores) | Predictor | F(1,151) | Sig |
| --- | --- | --- | --- |
| Hippocampus |  |  |  |
| Volume | Group | 14.7 | <.0001 |
|  | Age | 27.3 | <.0001 |
|  | Gender | 0.18 | .668 |
| T2μ | Group | 1.07 | .345 |
|  | Age | 2.28 | .133 |
|  | Gender | 2.50 | .116 |
| T2σ | Group | 10.8 | <.0001 |
|  | Age | 21.4 | <.0001 |
|  | Gender | 15.1 | .0002 |
| Thalamus |  |  |  |
| Volume | Group | 3.22 | .043 |
|  | Age | 17.1 | <.0001 |
|  | Gender | 1.83 | .179 |
| T2μ | Group | 2.65 | .074 |
|  | Age | 55.0 | <.0001 |
|  | Gender | 0.61 | .435 |
| T2σ | Group | 5.97 | .003 |
|  | Age | 17.0 | <.0001 |
|  | Gender | 6.00 | .015 |

### Further Discussion

#### Discrepancies between studies of T2 in humans and transgenic rodent models

Our proposed model (Figure 5) can begin to explain the discrepancy between studies of T2 in rodent models of Alzheimer’s disease and human cases. As summarised in a recent review (13), the majority of studies to date looking at T2 alterations in rodent models of Alzheimer’s disease observe decreased T2 in the hippocampus. However, in human literature the picture is much more varied, with most studies reporting a T2 increase (14-18), some reporting no change at all (or changes in just one hemisphere) (18, 19), and some reporting a decline (20, 21). Considering the currently presented model, this suggests that the animal models of Alzheimer’s disease used to test this effect have an over-dominance of T2-shortening factors. Given that these transgenic models are overwhelmingly based on mutations within amyloid cascade pathways, it is reasonable to conclude that these models may lead to a more dominant effect of Aβ on T2 in these models than is the case in “human” Alzheimer’s disease. Indeed, many transgenic rodent models express an abundance of Aβ plaques but show little or no neurodegeneration (22), and therefore have fewer regions of increased T2. Furthermore, most people with late-onset Alzheimer’s disease do not have mutations in any of the primary genes modified in rodent models. Many of these transgenic mouse models are models of amyloidopathy rather than models of Alzheimer’s disease; they do not necessarily reflect the heterogeneity seen in Alzheimer’s disease in humans.

#### Differences between T2 profiles in hippocampus vs thalamus

The overall pattern of results is very similar between hippocampus and thalamus. However, a primary difference is that age is a strong predictor of T2 midpoint only in the thalamus. By only looking at T2 midpoint, it is easy to conclude that the hippocampus is resistant to age and the thalamus is sensitive, as is concluded by Kirsch, Jacobs (15). However, when looking at the distribution of T2 (that is, T2 heterogeneity), it is clear that the hippocampus is not unaffected by age, but rather, factors that increase and decrease T2 are more evenly balanced than in the thalamus. Given that loci of amyloid deposition throughout the brains of both healthy and Alzheimer’s disease patients is similar (23, 24), Aβ is more likely to be found in the MTL than in the thalamus, even in individuals with no Alzheimer’s disease symptoms. This supports the idea that even in “healthy” hippocampi, some Aβ will be causing T2-shortening coincident with T2-lengthening factors dominant in aging, the latter effects only becoming dominant in much older people.

The effect size of group on T2 heterogeneity was stronger for hippocampal T2 than thalamic T2. These results reflect previous literature on the severity of histopathological changes in either region (25). Here, Braak, Braak (25) report that the thalamus, unlike the hippocampus, is relatively resistant to atrophy in Alzheimer’s disease until relatively late stages, despite early deposition of Aβ, and possibly as a result of later deposition of NFTs. As a result, the increase in T2 heterogeneity may be an early indicator of imminent neuronal loss. An alternate explanation is that early changes in T2 only occur in a small portion of the thalamus. Future work will explore individual hemisphere effects and subfields of both the thalamus and hippocampus, though this is beyond the scope of this paper.

Both of these differences support the use of T2 heterogeneity over T2 midpoint as a measure of tissue integrity.

#### T2 heterogeneity and volume were no different between MCI and AD groups

We observed no difference between MCI and AD groups in any structural measure. All conclusions comparing the AD group in this study directly against other groups must be drawn carefully. The relatively small sample size and the lack of a clear distinction between MCI and AD diagnostic criteria (primarily based on an assessment of whether cognitive impairment is significantly affecting daily living (26)) increase variation within the AD group. Additionally, automatic masking procedures did not contain AD data in training datasets (though the ASHS atlas does contain data from MCI patients for hippocampal segmentation) (27, 28). Priors in segmentation algorithms may therefore be biased in cases where atrophy is most severe, causing underestimation of volume loss in the AD group. Overestimation of the mask due to prior bias may cause overlap into regions of CSF which could also lead to the results we see with T2 relaxometry; a trend towards higher T2 midpoint in AD compared to MCI, but equal or smaller T2 heterogeneity. However, it is feasible that at later stages of the disease, when damage is more severe and a substantial proportion of voxels display increased T2 (Figure 6), T2 distribution width will plateau and may indeed begin to narrow due to clustering around a higher (pathological) midpoint.

#### References

1. Nasreddine ZS, Phillips NA, Bédirian V, Charbonneau S, Whitehead V, Collin I, et al. The Montreal Cognitive Assessment, MoCA: a brief screening tool for mild cognitive impairment. Journal of the American Geriatrics Society. 2005;53(4):695-9.

2. Strauss E, Sherman EMS, Spreen O. A Compendium of Neuropsychological Tests: administration, norms and commentary. 3 ed. New York: Oxford Univeristy Press; 2006.

3. Cambridge Cognition. CANTAB Insight: A Scientific and Clinical Review. Cambridge Cognition Limited; 2016.

4. Blackwell AD, Sahakian BJ, Vesey R, Semple JM, Robbins TW, Hodges JR. Detecting Dementia: Novel Neuropsychological Markers of Preclinical Alzheimer’s Disease. Dementia and Geriatric Cognitive Disorders. 2003;17(1-2):42-8.

5. Fowler KS, Saling MM, Conway EL, Semple JM, Louis WJ. Paired associate performance in the early detection of DAT. Journal of the International Neuropsychological Society. 2002;8(1):58-71.

6. Hsieh S, Schubert S, Hoon C, Mioshi E, Hodges JR. Validation of the Addenbrooke's Cognitive Examination III in Frontotemporal Dementia and Alzheimer's Disease. Dementia and Geriatric Cognitive Disorders. 2013;36(3-4):242-50.

7. Elwood RW. The California Verbal Learning Test: Psychometric characteristics and clinical application. Neuropsychology Review. 1995;5(3):173-201.

8. Strauss E, Sherman EMS, Spreen O. A compendium of neuropsychological tests: Administration, norms, and commentary. 3 ed. New York, NY, US: Oxford University Press; 2006.

9. Berg L. Clinical Dementia Rating (CDR). Psychopharmacology bulletin. 1988;24(4):637-9.

10. Wilson BA, Greenfield E, Clare L, Baddeley A, Cockburn J, Watson P, et al. The Rivermead Behavioural Memory Test - Third Edition. London, UK: Pearson; 2008.

11. Wilson B, Cockburn J, Baddeley A, Hiorns R. The development and validation of a test battery for detecting and monitoring everyday memory problems. Journal of Clinical and Experimental Neuropsychology. 1989;11(6):855-70.

12. Beardsall L, Huppert FA. A comparison of clinical, psychometric and behavioural memory tests: Findings from a community study of the early detection of dementia. International Journal of Geriatric Psychiatry. 1991;6(5):295-306.

13. Tang X, Cai F, Ding D-X, Zhang L-L, Cai X-Y, Fang Q. Magnetic resonance imaging relaxation time in Alzheimer’s disease. Brain research bulletin. 2018.

14. Raven EP, Lu PH, Tishler TA, Heydari P, Bartzokis G. Increased Iron Levels and Decreased Tissue Integrity in Hippocampus of Alzheimer's Disease Detected in vivo with Magnetic Resonance Imaging. Journal of Alzheimer's Disease. 2013;37(1):127-36-36.

15. Kirsch SJ, Jacobs RW, Butcher LL, Beatty J. Prolongation of magnetic resonance T2 time in hippocampus of human patients marks the presence and severity of Alzheimer's disease. Neuroscience Letters. 1992;134(2):187-90.

16. Pitkanen A, Laakso M, Kalviainen R, Partanen K, Vainio P, Lehtovirta M, et al. Severity of hippocampal atrophy correlates with the prolongation of MRI T sub 2 relaxation time in temporal lobe epilepsy but not in Alzheimer's disease. Neurology. 1996;46(6):1724-30.

17. Wang H, Yuan H, Shu L, Xie J, Zhang D. Prolongation of T2 relaxation times of hippocampus and amygdala in Alzheimer's disease. Neuroscience Letters. 2004;363(2):150-3.

18. Laakso MP, Partanen K, Soininen H, Lehtovirta M, Hallikainen M, Hänninen T, et al. MR T2 relaxometry in Alzheimer's disease and age-associated memory impairment. Neurobiology of Aging. 1996;17(4):535-40.

19. Campeau NG, Petersen RC, Felmlee JP, O'Brien PC, Jack CR. Hippocampal transverse relaxation times in patients with Alzheimer disease. Radiology. 1997;205(1):197-201-.

20. House MJ, Pierre STG, Foster JK, Martins RN, Clarnette R. Quantitative MR imaging R2 relaxometry in elderly participants reporting memory loss. AJNR American journal of neuroradiology. 2006;27(2):430-9.

21. Luo Z, Zhuang X, Kumar D, Wu X, Yue C, Han C, et al. The Correlation of Hippocampal T2-Mapping with Neuropsychology Test in Patients with Alzheimer’s Disease. PLoS ONE. 2013;8(9).

22. Philipson O, Lord A, Gumucio A, O’Callaghan P, Lannfelt L, Nilsson LNG. Animal models of amyloid‐β‐related pathologies in Alzheimer’s disease. FEBS Journal. 2010;277(6):1389-409.

23. Thal DR, Rüb U, Orantes M, Braak H. Phases of A-Beta-deposition in the human brain and its relevance for the development of AD. Neurology. 2002;58(12):1791-800.

24. Arriagada PV, Marzloff K, Hyman BT. Distribution of Alzheimer-type pathologic changes in nondemented elderly individuals matches the pattern in Alzheimer's disease. Neurology. 1992;42(9):1681-.

25. Braak H, Braak E, Yilmazer D, de Vos RAI, Jansen ENH, Bohl J. Pattern of brain destruction in Parkinson's and Alzheimer's diseases. Journal of Neural Transmission. 1996;103(4):455-90-90.

26. Albert MS, DeKosky ST, Dickson D, Dubois B, Feldman HH, Fox NC, et al. The diagnosis of mild cognitive impairment due to Alzheimer’s disease: Recommendations from the National Institute on Aging-Alzheimer’s Association workgroups on diagnostic guidelines for Alzheimer's disease. Alzheimer's & Dementia. 2011;7(3):270-9.

27. Yushkevich PA, Pluta JB, Wang H, Xie L. Automated volumetry and regional thickness analysis of hippocampal subfields and medial temporal cortical structures in mild cognitive impairment. Human Brain Mapping. 2015;36(1):256-87.

28. Iglesias JE, Augustinack JC, Nguyen K, Player CM, Player A, Wright M, et al. A computational atlas of the hippocampal formation using ex vivo, ultra-high resolution MRI: Application to adaptive segmentation of in vivo MRI. NeuroImage. 2015;115(Prog. Brain Res. 163 2007):117-37.
